# Swimming and the human microbiome at the intersection of sports, clinical, and environmental sciences: A scoping review of the literature

**DOI:** 10.3389/fmicb.2022.984867

**Published:** 2022-08-03

**Authors:** Luca Puce, Jarrad Hampton-Marcell, Khaled Trabelsi, Achraf Ammar, Hamdi Chtourou, Ayoub Boulares, Lucio Marinelli, Laura Mori, Filippo Cotellessa, Antonio Currà, Carlo Trompetto, Nicola Luigi Bragazzi

**Affiliations:** ^1^Department of Neuroscience, Rehabilitation, Ophthalmology, Genetics, Maternal and Child Health (DINOGMI), University of Genoa, Genoa, Italy; ^2^Department of Biological Sciences, University of Illinois at Chicago, Chicago, IL, United States; ^3^Biosciences Division, Argonne National Laboratory, Lemont, IL, United States; ^4^Institut Supérieur du Sport et de l’Éducation Physique de Sfax, Université de Sfax, Sfax, Tunisia; ^5^Research Laboratory: Education, Motricité, Sport et Santé, EM2S, Sfax University, Sfax, Tunisia; ^6^Department of Training and Movement Science, Institute of Sport Science, Johannes Gutenberg-University Mainz, Mainz, Germany; ^7^Institute of Sport Science, Otto-von-Guericke University Magdeburg, Magdeburg, Germany; ^8^Interdisciplinary Laboratory in Neurosciences, Physiology and Psychology: Physical Activity, Health and Learning (LINP2), Université Paris Lumières, Paris Nanterre University, Nanterre, France; ^9^Activité Physique, Sport et Santé, UR18JS01, Observatoire National du Sport, Tunis, Tunisia; ^10^Higher Institute of Sports and Physical Education of Ksar-Said, University of Manouba, Tunis, Tunisia; ^11^Istituto di Ricovero e Cura a Carattere Scientifico (IRCCS) Ospedale Policlinico San Martino, Genoa, Italy; ^12^Department of Medical-Surgical Sciences and Biotechnologies, A. Fiorini Hospital, Sapienza University of Rome, Latina, Italy; ^13^Laboratory for Industrial and Applied Mathematics, Department of Mathematics and Statistics, York University, Toronto, ON, Canada

**Keywords:** microbiome, swimming, sports microbiomics, clinical microbiomics, scoping review

## Abstract

The human microbiota is comprised of more than 10–100 trillion microbial *taxa* and symbiotic cells. Two major human sites that are host to microbial communities are the gut and the skin. Physical exercise has favorable effects on the structure of human microbiota and metabolite production in sedentary subjects. Recently, the concept of “athletic microbiome” has been introduced. To the best of our knowledge, there exists no review specifically addressing the potential role of microbiomics for swimmers, since each sports discipline requires a specific set of techniques, training protocols, and interactions with the athletic infrastructure/facility. Therefore, to fill in this gap, the present scoping review was undertaken. Four studies were included, three focusing on the gut microbiome, and one addressing the skin microbiome. It was found that several exercise-related variables, such as training volume/intensity, impact the athlete’s microbiome, and specifically the non-core/peripheral microbiome, in terms of its architecture/composition, richness, and diversity. Swimming-related power-/sprint- and endurance-oriented activities, acute bouts and chronic exercise, anaerobic/aerobic energy systems have a differential impact on the athlete’s microbiome. Therefore, their microbiome can be utilized for different purposes, including talent identification, monitoring the effects of training methodologies, and devising *ad hoc* conditioning protocols, including dietary supplementation. Microbiomics can be exploited also for clinical purposes, assessing the effects of exposure to swimming pools and developing potential pharmacological strategies to counteract the insurgence of skin infections/inflammation, including acne. In conclusion, microbiomics appears to be a promising tool, even though current research is still limited, warranting, as such, further studies.

## Introduction

The human microbiota is comprised of more than 10–100 trillion microbial (bacterial, and non-bacterial, such as archaeal, viral, fungal, eukaryal, and parasitical) *taxa* and symbiotic cells (Ursell et al., 2012), the majority of which reside in the gut (Thursby and Juge, 2017). The human microbiome, a term coined by Dr. Joshua Lederberg in 2001, is the comprehensive catalog of genes harbored by these microbial communities (Lederberg and McCray, 2001; Liu, 2016): more than three million genes constitute the intestinal microbiome. Reflecting the mixture of microbes and the diversity of the microbial ecosystem, this consists of several components or compartments (Matijašic et al., 2020): namely, the bacteriome (Donaldson et al., 2016), the archaeome (Borrel et al., 2020), the virome (Liang and Bushman, 2021), the mycobiome (Chin et al., 2020), the eukaryome (Hamad et al., 2016), and the parasitome (Marzano et al., 2017).

Two major human sites that are host to microbial communities are the gut and the skin (De Pessemier et al., 2021). Both microbiomes are extremely heterogeneous, dynamic, and plastic, consisting of a highly diverse population of microbes that can have both beneficial and detrimental impacts on human health (Ogunrinola et al., 2020). In particular, the gut microbiome is composed of more than 1,200 species of bacteria (Jandhyala et al., 2015), including *Bacteroides*, *Actinomycetes*, *Firmicutes*, *Proteobacteria*, and *Verrucomicrobia*. It plays different immunometabolic functions, ranging from nutrient absorption (in particular, micro-nutrient uptake), and processing to vitamin synthesis, energy harvest, and metabolic homeostasis (including promoting and favoring insulin sensitivity), and fine-tuning/modulation of the immune system and of the inflammatory response at the host level, protecting especially newborns from respiratory and intestinal infections and pathogen invasion (Belkaid and Hand, 2014). It can also provide the individual with sources of energy, by fermenting and processing short-chain fatty acids (SCFAs), like butyrate, acetate, and propionate (den Besten et al., 2013; Portincasa et al., 2022).

The skin microbiome is complex, dynamic, and heterogeneous as well (Stacy and Belkaid, 2019). Skin represents the body’s first line of defense against invading microorganisms. The skin microbiome has been shown to provide immunity against exogenous bacterial colonization (Byrd et al., 2018). Some environmental (terrestrial, marine, and freshwater) exposures, including, for instance, recreational water exposures, may alter the skin microbiome and potentially induce skin infections (Nielsen and Jiang, 2019; Patra et al., 2020).

Physical exercise has favorable effects on the structure of gut microbiota and metabolite production in sedentary subjects (Cella et al., 2021; Clauss et al., 2021). The body of currently available evidence is mostly from animal studies: microbial community architecture has been found to exert beneficial effects in terms of microbial composition, structure, richness, and diversity, favoring and promoting the establishment of commensal bacteria, and an anti-inflammatory *milieu* and counteracting/mitigating against pro-inflammatory effects, and optimizing performance-related outcomes. Moreover, it can interact with diet and other lifestyles to further enhance performance (Donati Zeppa et al., 2019; Cella et al., 2021). Of note, alterations in the microbiome can also be a consequence of sports and physical activity (such as swimming) (Barton et al., 2018; Mohr et al., 2020).

Recently, the concept of “athletic microbiome” (Barton et al., 2018; Mohr et al., 2020) has been introduced. Whereas some reviews have synthesized current state-of-art concerning endurance exercise (Mach and Fuster-Botella, 2017) and competitive sports (Wegierska et al., 2022), to the best of our knowledge, there exists no review specifically addressing the potential role of microbiomics for swimmers, since each sports discipline requires a specific set of techniques, training protocols, and interactions with the athletic infrastructure/facility (in this case, the swimming pool) (Xu et al., 2022). Research has shown that swimming can exert a plethora of regulatory effects on the microbiome, in terms of immunometabolic and neuroimmunological ones, as demonstrated by a number of animal studies (Huang et al., 2019; Xie et al., 2022). However, little is known about the impact of training protocols on the microbiome among swimmers and whether adjustments in an athletic program impact overall changes in the gut microbiome in swimmers, with a particular focus on high-level/elite athletes. Also, there is a lack of prospective, longitudinal studies on the temporal changes and trends at the microbiome level. Therefore, to fill in this gap of knowledge, the present scoping review was undertaken.

## Materials and methods

We devised the present review as scoping review, in that the research question was broad and intersectional, across several disciplines (sports sciences, microbiology, biotechnology, and molecular biology). A scoping review is an innovative technique to rapidly synthesize and map the literature on a designated topic in terms of major concepts, sources, and types of evidence (Arksey and O’Malley, 2005; Khalil and Tricco, 2022; Pollock et al., 2022). Several methodologies and guidelines exist: in particular, we leveraged Arksey and O’Malley’s six-stage approach (Arksey and O’Malley, 2005). Firstly, we identified the research question and we built and developed our multidisciplinary team. We used the “population/participants-concepts-context” (PCC) mnemonic. “Population/participants” were athletes of any competitive level, national or international, short- or long-distance, and the main concept was about the potential applications of microbiomics within this specific sports discipline. The “context” was worldwide (our search was not confined to a particular territory/geographic location). Based on a preliminary literature search, an *a priori* protocol was devised. MEDLINE, a major scholarly, electronic biomedical database, was accessed *via* PubMed, a freely available interface. No time or language restrictions were applied. The search string consisted of two major components: microbiome and swimmers, with synonyms/variants properly linked by using Boolean operators [(microbiome OR microbiota OR “bacterial community” OR “bacterial communities” OR “bacterial flora”) AND (swimming OR swimmer)]. “Medical subject headings” (MeSH) terms and wild-card (truncated words) options were used. Extensive cross-referencing was carried out. Further, specific target journals were hand-searched. Moreover, also gray literature was consulted, by mining Google Scholar. Then, studies were selected for inclusion based on pre-specified inclusion and exclusion criteria, which were formulated based both on the PCC mnemonic and the “population/participants-intervention-comparator/comparison-outcome-study design” (PICOS) components. Studies were included if focusing on a population of swimmers (P), of any competitive level, subjected to a particular training protocol (I). Studies were deemed eligible if comparing swimmers against the general population. Other comparisons of interest included gender- and age-specific comparisons or related to a particular swimming style (C). Outcomes of interest were the quantification of the changes in the microbiome, in terms of architecture/composition, richness, or diversity (O) (see Tables 1, 2). Any study design was eligible for inclusion: retrospective, prospective, quantitative, observational, interventional, randomized, or non-randomized (S). Included studies were synthesized in a narrative fashion. Major topics/themes were identified by means of thematic analysis and overviewed qualitatively. Furthermore, we followed the “Preferred Reporting Items for Systematic Reviews and Meta-Analyses” (PRISMA) extension for scoping reviews (PRISMA-Scr) (Tricco et al., 2018). Finally, a formal quality appraisal was not conducted given that is not a mandatory component of scoping reviews.

**TABLE 1:** Microbiome-related terms/expressions.

Microbiome-related term/expression	Explanation
Richness	The total number of microbial species in a given microbiome
Diversity	The amount of individual microbes from each species present in a given microbiome
Alpha diversity	A measure of microbiome diversity related to a single sample (within-sample diversity)
Beta diversity	A measure of similarity/dissimilarity of different communities/populations (between-sample diversity)

**TABLE 2:** Search strategy adopted in the present scoping review.

Search strategy component	Related item(s)
Searched databases	PubMed/MEDLINE, Google scholar
Search string used	(microbiome OR microbiota OR “bacterial community” OR “bacterial communities” OR “bacterial flora”) AND (swimming OR swimmer)
Inclusion criteria	PCC
	Population/participants: athletes of any competitive level, national or international, short- or long-distance
	Concept: the potential applications of microbiomics within the sports discipline of swimming
	Context: worldwide
	PICOS
	Population/participants: swimmers, of any competitive level
	Intervention: any training protocol
	Comparator/comparison: swimmers against the general population; gender- and age-specific comparisons or comparison related to a particular swimming style
	Outcome(s): quantification of the changes in the microbiome, in terms of architecture/composition, richness, or diversity
	Study design: any study design (retrospective, prospective, quantitative, observational, interventional, randomized, or non-randomized)
Time restriction	None applied
Language filter	None applied

## Results

The initial search yielded 195 items. One hundred eighty-six studies were discarded after reading the title and/or the abstract, as they were irrelevant to the topic under study. Nine studies were screened in full text. Five studies were excluded with reason, since they did not meet our PICOS criteria (the population consisted of non-athletes). Finally, four studies were included in the present scoping review. Three of them focused on the gut microbiome, and one addressed the skin microbiome.

### Sports microbiomics in swimmers: Effects of training and probiotic consumption

Bielik et al. (2022) sampled from a longitudinal prospective study and recruited 17 and 7 young competitive male and female swimmers, respectively, aged 16–25 years. The authors assessed the impact of a 7-week, high-intensity training (HIT) program with or without probiotic (*Bryndza* sheep-cheese) consumption (30 g, 3–4 times *per* week) on swimming performance-related outcomes during the Slovak Swimming National Championship over a long course (being the pool 50 m in length). The probiotic contains 3 microbial families, 24 *genera*, and 44 species. Total DNA was extracted from stool samples and amplified utilizing primers that specifically target the V1-V3 regions of 16SrDNA. 300 bp pair-end reads were obtained, collected, and processed. The HIT program was comprised of swimming lengths of 12.5, 25, 50, and 100 m, carried out at an intensity of > 90% of maximum speed. The authors were able to find a HIT-induced increase in alpha diversity [in terms of operational taxonomic units (OTUs), Shannon index, but not Simpson index], independently of probiotic consumption. In particular, in the HIT group, among the most represented *phyla*, *Firmicutes* decreased from 80.2 to 76.3%, whereas *Bacteroidota* and *Actinobacteriota* increased from 17.7 to 21.6% and from 0.99 to 1%. In the HIT + probiotic consumption (HITB), *Firmicutes* and *Actinobacteriota* decreased from 82.3 to 77.7% and from 2.1 to 1.1%, respectively, whilst *Bacteroidota* increased from 14.1 to 19.9%. The *phyla Proteobacteria*, *Verrucomicrobiota*, *Cyanobacteria*, *Desulfobacterota*, *Fusobacteriota*, *Fibrobacterota*, *Patescibacteria*, and *Campylobacterota* were detected with an abundance lower than 1% in the HIT group. Similarly, in the HITB group, these *phyla* (with the exception of *Fusobacteriota*, and *Fibrobacterota*) could be reported. In terms of families, the *Lachnospiraceae* family was abundant both in the HIT and HITB groups. It was found to increase in the former group (from 41.5 to 43.5%) and to decrease in the latter (from 47.6 to 45.4%). Other abundant families in both groups were *Ruminococcaceae*, *Bacteroidaceae*, *Prevotellaceae*, and *Oscillospiraceae*. Furthermore, in terms of *genera*, *Faecalibacterium*, *Blautia*, *Bacteroides*, *Roseburia*, *Subdoligranulum*, *Ruminococcus*, *Prevotella*_9, *Agathobacter*, *Coprococcus*, and the *Ruminococcus torques* group could be identified in both groups. In terms of statistical significance, *Bacteroidiota* increased in both groups (*p* = 0.005 in HIT, *p* = 0.0260 in HITB). Concerning lactic acid bacteria, the order *Lactobacillales* (*p* = 0.015) and the family *Streptococcaceae* (*p* = 0.019) were significantly different pre vs. post in the HITB group. *Lactococcus* spp. was found to be increased in both groups (*p* = 0.046 in HIT, *p* = 0.008 in HITB), with a higher effect size in the probiotic consumers (12.8-fold vs. 5-fold change). The increase in HIT was reflected in the increase in anaerobic metabolism (namely, increased concentrations of pyruvate, and lactate, and decreased levels of acetate, and butyrate) as well as in the increase of bacterial species producing SCFA metabolites, such as *Butyricimonas* (*p* = 0.028) and *Alistipes* (*p* = 0.010). The latter increased also in the HITB group, but only in a borderline fashion (*p* = 0.060). Finally, by means of a machine-learning approach (random forest), the authors were able to build a set of parameters (acetate, pyruvate, *Butyricimonas*, butyrate, *Bacteroidetes*, *Alistipes*, and α-diversity measured by means of the Shannon index; pyruvate, lactate, acetate, α-diversity/Shannon index, and butyrate) able to differentiate pre- vs. post-intervention in HIT and HITB, respectively, with Area under the Curve (AUC) values of 0.78 and 0.99.

### Sports microbiomics in swimmers: Effects of detraining

Hampton-Marcell et al. (2020) recruited a sample of 13 (8 women and 5 men) collegiate swimmers aged 18–24 years from a Division 1 university. Microbial community small-subunit (SSU) rRNA genes were amplified using barcoded PCR primers targeting the V4 region and barcoded SSU rRNA amplicons were, subsequently, cleaned and processed. 150 nt sequences were obtained from the pooled DNA, and 79 samples were collected, totaling 395,000 16S rRNA sequences and 7,684 OTUs. The most abundant bacterial *phyla* were *Bacteroidetes* (46.5%) and *Firmicutes* (46.6%) *phyla*, with an average ratio of *Firmicutes*: *Bacteroidetes* of 2:1 at the peak of the training program. The most represented families were *Bacteroidaceae* (39.5%), *Lachnospiraceae* (16.6%), and *Ruminococcaceae* (14.0%) over the entire study period. *Porphyromonas* (9.2%), *Sutterella* (7.9%), and unclassified *genera* within the families *Lachnospiraceae* and *Ruminococcaceae* (5.8%) were identified as the commonest *taxa*. Whilst no differences in terms of body composition and anthropometric measurements (fat mass, fat-free mass, or weight) could be computed, in terms of Bray-Curtis dissimilarity between study training phases, microbial community diversity and structure were impacted by changes in training volume and shifted 43% on average. Along with changes in beta diversity, alpha diversity changed too, positively correlating with yardage *per* week, decreasing and paralleling decreases in training volume, as quantitatively assessed utilizing both the Shannon index and community evenness (the inverse Simpson index). This ratio gradually decreased to 1:1, with the decreases in training. Detraining was reflected in reduced energy harvesting and expenditure/consumption by *Firmicutes*-derived microbes. A “core” component of the microbiome could be identified, with 82% of the OTUs being consistent over time and the different study phases, and being similar among the swimmers. Finally, two major families (*Lachnospiraceae* and *Ruminococcaceae*), and two major *genera* (*Coprococcus* and *Faecalibacterium*) robustly correlated with short-term changes in training volume.

### Genetic and allelic regulation and sports microbiomics in swimmers: Correlations with performance outcomes

The GALANTL6 gene, at 4q34.1, consists of 21 exons and is expressed mainly in adult testis, brain, spinal cord, and cerebellum, as well as at the level of the skeletal muscle tissue. It encodes the enzyme polypeptide N-acetylgalactosaminyltransferase like type 6, which plays a key role in the metabolic homeostasis (specifically of lactate) and regulation of the gut microbiota *via* O-glycosylation and degradation of glycans. In particular, the gene can modulate the cycle (synthesis and resynthesis) and properties (anti-inflammatory effects) of the microbial species processing and producing SCFAs. Li et al. (2015) and Zmijewski et al. (2021) assessed the hypothesis that the T allele of the GALNTL6 rs558129 single-nucleotide polymorphism (SNP) could positively impact anaerobic metabolism and athletic performance in a sample of 147 Polish short-distance and 49 long-distance swimmers, taking part into national or international competitions. These elite athletes (aged 20.31 ± 2.67 years) were matched with 379 sedentary students, acting as controls (aged 22.6 ± 2.8 years). The study cohort was genotyped using the real-time polymerase chain reaction (real-time PCR). The SNP was in Hardy-Weinberg *equilibrium* in controls and in the entire study population. When compared to their CC homozygote counterparts, carriers of the T allele (CT + TT) displayed a 1.56 times higher likelihood of being short-distance swimmers. There was an overrepresentation of the T allele among swimmers, even though this correlation did not achieve statistical significance in long-distance swimmers. Finally, no significant effect of sex and gender could be computed.

### Clinical microbiomics in swimmers

*Cutibacterium acnes* (*C. acnes*, formerly known as *Propionibacterium acnes*) is an opportunistic pathogen that plays a major role in the etiopathogenesis of acne. Swimmers should be protected against this dermatological disease, in that they regularly have immersion in antimicrobial chlorine, even though some reports have shown that chlorine in the pools can affect the swimmer’s microbiome and metabolome (van Veldhoven et al., 2018; Morss-Walton et al., 2022). However, it is a commonly reported clinical observation that adolescent swimmers may suffer from acne and even develop standard therapies-resistant acne. Besides some potential mechanisms (such as skin dryness, and impaired barrier function) that can be hypothesized, another driver of the so-called “swimmer’s acne” could be the presence of microorganisms, such as the family *Pseudomonadaceae* (a family of gram-negative *bacteria*, including *Pseudomonas aeruginosa*), associated with recreational water, hot tubs, and swimming facilities. Morss-Walton et al. (2022) investigated the microbial dynamics of *C. acnes* and *Pseudomonadaceae* pre- vs. post-swimming in a sample of 16 swimmers (8 girls and 8 boys, 75% whites), belonging to a local competitive swimming club, seven of which suffering from acne. Coproporphyrin III (CPIII), the main porphyrin produced by *C. acnes*, was measured by means of fluorescence photography to quantify the absolute abundance of the pathogen on the face of each participant. The technique of 16S rRNA gene sequencing using primers targeting the V4 region was exploited to characterize the skin microbiome, after the collection of skin swabs. CPIII fluorescence levels were found to be reduced after 1 h of swimming (*p*-value < 0.001), whereas the relative abundances of *C. acnes* and of *Pseudomonadaceae* were stable (slightly increasing from 15.0 to 19.0%) and increased (*p* = 0.027, from 0.4 to 1.7%), respectively. The relative abundances of *Gemellales*, *Lactobacillales*, *Pasteurellales*, *Pasteurellaceae*, *Streptococcus*, and *Lautropia* significantly decreased. Of note, after swimming, alpha diversity of the skin microbiome decreased in terms of the Shannon index, the Chao1 index, and observed OTUs (*p*-value < 0.001 for all three metrics). On the contrary, beta diversity (in terms of the OTU Bray-Curtis distance) increased after swimming. In conclusion, the authors found that decolonization and colonization of *C. acnes* and *Pseudomonadaceae* may result in skin dysbiosis and acne.

## Discussion and conclusion

Microbiomics represents an emerging field (Neu et al., 2021), with increasing applications in the sports arena. Microbial metrics can well characterize an athlete’s energy utilization, even when changes in physical activity levels and adjustments of training protocols do not reflect in biochemical (such as total cholesterol, insulin, or glucose) (Bielik et al., 2022), body composition and anthropometric (like fat mass, fat-free mass, or weight), or fitness measures (Hampton-Marcell et al., 2020). The human microbiome is an excellent predictor of changes in host phenotype and, more generally speaking, in phenome (Ursell et al., 2012; Neu et al., 2021), explaining up to 20% of host adaptation and related cellular/molecular phenomena, whilst the genome can explain up to less than 2% of host-related modifications.

Comprehensive sophisticated approaches, including high-throughput quantitative polymerase chain reaction (qPCR)/real-time PCR, amplicon and shotgun genomic DNA sequencing, as well as 16S rRNA gene sequencing, can be exploited to thoroughly characterize the human microbiome in athletes (Han et al., 2020).

Whereas 70–80% of the microbiome (defined as the “core microbiome”) remains stable over time, the so-called non-core or peripheral microbiome is susceptible to environmental/external *stimuli* and exposures. A “core microbiome” can be defined as “any set of microbial *taxa*, or the genomic and functional attributes associated with those *taxa*, that are characteristic of a host or environment of interest” (Neu et al., 2021).

Several exercise-related variables, such as training volume/intensity, impact the athlete’s microbiome, and specifically the non-core/peripheral microbiome, in terms of its architecture, composition, richness, and diversity. Swimming-related power-/sprint- and endurance-oriented activities, acute bouts, and chronic exercise, anaerobic and aerobic energy systems have a differential impact on the athlete’s microbiome, specifically in the swimmers (Li et al., 2015; Hampton-Marcell et al., 2020; Zmijewski et al., 2021; Bielik et al., 2022). Therefore, their microbiome can be utilized for different purposes, including talent identification, monitoring the effects of training methodologies, and devising *ad hoc* conditioning protocols, including the administration of supplements and probiotics.

Moreover, given the marked inter-individual variability in microbial changes and shifts, microbiomics could be a valuable tool to monitor athletes’ response to exercise and diet, personalizing training protocol as well as sports nutrition to enhance performance-related outcomes (Hughes, 2020; Sorrenti et al., 2020; Hughes and Holscher, 2021). Microbiomics can be exploited also for clinical purposes, assessing the effects of exposure to water facilities (swimming pools) and developing potential pharmacological strategies to counteract the insurgence of skin infections and inflammation, including acne.

In conclusion, microbiomics appears to be a promising tool to investigate the impact of training, detraining, dietary intake and supplements/probiotics use among swimmers, as well as clinical effects of interactions with swimming facilities, even though current research is still limited, warranting, as such, further studies.

## Author contributions

All authors listed have made a substantial, direct, and intellectual contribution to the work, and approved it for publication.

## References

[B1] Arksey H. O’Malley L. (2005). Scoping Studies: Towards a Methodological Framework. *Int. J. Soc. Res. Methodol.* 8 19–32. 10.1080/1364557032000119616

[B2] Barton W. Penney N. C. Cronin O. Garcia-Perez I. Molloy M. G. Holmes E. (2018). The microbiome of professional athletes differs from that of more sedentary subjects in composition and particularly at the functional metabolic level. *Gut* 67 625–633. 10.1136/gutjnl-2016-313627 28360096

[B3] Belkaid Y. Hand T. W. (2014). Role of the microbiota in immunity and inflammation. *Cell* 157 121–141. 10.1016/j.cell.2014.03.011 24679531 PMC4056765

[B4] Bielik V. Hric I. Ugrayová S. Kubáňová L. Putala M. Grznár Ĺ (2022). Effect of High-intensity Training and Probiotics on Gut Microbiota Diversity in Competitive Swimmers: Randomized Controlled Trial. *Sports Med. Open* 8:64. 10.1186/s40798-022-00453-8 35536489 PMC9091066

[B5] Borrel G. Brugère J. F. Gribaldo S. Schmitz R. A. Moissl-Eichinger C. (2020). The host-associated archaeome. *Nat. Rev. Microbiol.* 18 622–636. 10.1038/s41579-020-0407-y 32690877

[B6] Byrd A. L. Belkaid Y. Segre J. A. (2018). The human skin microbiome. *Nat. Rev. Microbiol.* 16 143–155. 10.1038/nrmicro.2017.157 29332945

[B7] Cella V. Bimonte V. M. Sabato C. Paoli A. Baldari C. Campanella M. (2021). Nutrition and Physical Activity-Induced Changes in Gut Microbiota: Possible Implications for Human Health and Athletic Performance. *Foods* 10:3075. 10.3390/foods10123075 34945630 PMC8700881

[B8] Chin V. K. Yong V. C. Chong P. P. Amin Nordin S. Basir R. Abdullah M. (2020). Mycobiome in the Gut: A Multiperspective Review. *Mediators Inflamm.* 2020:9560684. 10.1155/2020/9560684 32322167 PMC7160717

[B9] Clauss M. Gérard P. Mosca A. Leclerc M. (2021). Interplay Between Exercise and Gut Microbiome in the Context of Human Health and Performance. *Front. Nutr.* 8:637010. 10.3389/fnut.2021.637010 34179053 PMC8222532

[B10] De Pessemier B. Grine L. Debaere M. Maes A. Paetzold B. Callewaert C. (2021). Gut-Skin Axis: Current Knowledge of the Interrelationship between Microbial Dysbiosis and Skin Conditions. *Microorganisms* 9:353. 10.3390/microorganisms9020353 33670115 PMC7916842

[B11] den Besten G. van Eunen K. Groen A. K. Venema K. Reijngoud D. J. Bakker B. M. (2013). The role of short-chain fatty acids in the interplay between diet, gut microbiota, and host energy metabolism. *J. Lipid Res.* 54 2325–2340. 10.1194/jlr.R036012 23821742 PMC3735932

[B12] Donaldson G. P. Lee S. M. Mazmanian S. K. (2016). Gut biogeography of the bacterial microbiota. *Nat. Rev. Microbiol.* 14 20–32. 10.1038/nrmicro3552 26499895 PMC4837114

[B13] Donati Zeppa S. Agostini D. Gervasi M. Annibalini G. Amatori S. Ferrini F. (2019). Mutual Interactions among Exercise, Sport Supplements and Microbiota. *Nutrients* 12:17. 10.3390/nu12010017 31861755 PMC7019274

[B14] Hamad I. Raoult D. Bittar F. (2016). Repertory of eukaryotes (eukaryome) in the human gastrointestinal tract: Taxonomy and detection methods. *Parasite Immunol.* 38 12–36. 10.1111/pim.12284 26434599

[B15] Hampton-Marcell J. T. Eshoo T. W. Cook M. D. Gilbert J. A. Horswill C. A. Poretsky R. (2020). Comparative Analysis of Gut Microbiota Following Changes in Training Volume Among Swimmers. *Int. J. Sports Med.* 41 292–299. 10.1055/a-1079-5450 31975357

[B16] Han M. Yang K. Yang P. Zhong C. Chen C. Wang S. (2020). Stratification of athletes’ gut microbiota: The multifaceted hubs associated with dietary factors, physical characteristics and performance. *Gut Microbes* 12 1–18. 10.1080/19490976.2020.1842991 33289609 PMC7734118

[B17] Huang W. C. Chen Y. H. Chuang H. L. Chiu C. C. Huang C. C. (2019). Investigation of the Effects of Microbiota on Exercise Physiological Adaption, Performance, and Energy Utilization Using a Gnotobiotic Animal Model. *Front. Microbiol.* 10:1906. 10.3389/fmicb.2019.01906 31551939 PMC6736621

[B18] Hughes R. L. Holscher H. D. (2021). Fueling Gut Microbes: A Review of the Interaction between Diet, Exercise, and the Gut Microbiota in Athletes. *Adv. Nutr.* 12 2190–2215. 10.1093/advances/nmab077 34229348 PMC8634498

[B19] Hughes R. L. A. (2020). Review of the Role of the Gut Microbiome in Personalized Sports Nutrition. *Front. Nutr.* 6:191. 10.3389/fnut.2019.00191 31998739 PMC6966970

[B20] Jandhyala S. M. Talukdar R. Subramanyam C. Vuyyuru H. Sasikala M. Nageshwar Reddy D. (2015). Role of the normal gut microbiota. *World J. Gastroenterol.* 21 8787–8803. 10.3748/wjg.v21.i29.8787 26269668 PMC4528021

[B21] Khalil H. Tricco A. C. (2022). Differentiating between mapping reviews and scoping reviews in the evidence synthesis ecosystem. *J. Clin. Epidemiol.* [Epub ahead of print]. 10.1016/j.jclinepi.2022.05.012 35636593

[B22] Lederberg J. McCray A. (2001). Ome sweet ’omics: – A genealogical treasury of words. *Scientist* 15:8.

[B23] Li J. H. Wang Z. H. Zhu X. J. Deng Z. H. Cai C. X. Qiu L. Q. (2015). Health effects from swimming training in chlorinated pools and the corresponding metabolic stress pathways. *PLoS One* 10:e0119241. 10.1371/journal.pone.0119241 25742134 PMC4351252

[B24] Liang G. Bushman F. D. (2021). The human virome: Assembly, composition and host interactions. *Nat. Rev. Microbiol.* 19 514–527. 10.1038/s41579-021-00536-5 33785903 PMC8008777

[B25] Liu X. (2016). Microbiome. *Yale J. Biol. Med.* 89 275–276.

[B26] Mach N. Fuster-Botella D. (2017). Endurance exercise and gut microbiota: A review. *J. Sport Health Sci.* 6 179–197. 10.1016/j.jshs.2016.05.001 30356594 PMC6188999

[B27] Marzano V. Mancinelli L. Bracaglia G. Del Chierico F. Vernocchi P. Di Girolamo F. (2017). Omic” investigations of protozoa and worms for a deeper understanding of the human gut “parasitome. *PLoS Negl. Trop. Dis.* 11:e0005916. 10.1371/journal.pntd.0005916 29095820 PMC5667730

[B28] Matijašić M. Meštrović T. Paljetak H. Č Perić M. Barešić A. Verbanac D. (2020). Gut Microbiota beyond Bacteria-Mycobiome, Virome, Archaeome, and Eukaryotic Parasites in IBD. *Int. J. Mol. Sci.* 21:2668. 10.3390/ijms21082668 32290414 PMC7215374

[B29] Mohr A. E. Jäger R. Carpenter K. C. Kerksick C. M. Purpura M. Townsend J. R. (2020). The athletic gut microbiota. *J. Int. Soc. Sports Nutr.* 17:24. 10.1186/s12970-020-00353-w 32398103 PMC7218537

[B30] Morss-Walton P. C. McGee J. S. Rosales Santillan M. Kimball R. Cukras A. Patwardhan S. V. (2022). Yin and Yang of skin microbiota in “swimmer acne”. *Exp. Dermatol.* 31 899–905. 10.1111/exd.14535 35118730

[B31] Neu A. T. Allen E. E. Roy K. (2021). Defining and quantifying the core microbiome: Challenges and prospects. *Proc. Natl. Acad. Sci. U. S. A.* 118:e2104429118. 10.1073/pnas.2104429118 34862327 PMC8713806

[B32] Nielsen M. C. Jiang S. C. (2019). Alterations of the human skin microbiome after ocean water exposure. *Mar. Pollut. Bull.* 145 595–603. 10.1016/j.marpolbul.2019.06.047 31590829 PMC8061468

[B33] Ogunrinola G. A. Oyewale J. O. Oshamika O. O. Olasehinde G. I. (2020). The Human Microbiome and Its Impacts on Health. *Int. J. Microbiol.* 2020:8045646. 10.1155/2020/8045646 32612660 PMC7306068

[B34] Patra V. Gallais Sérézal I. Wolf P. (2020). Potential of Skin Microbiome, Pro- and/or Pre-Biotics to Affect Local Cutaneous Responses to UV Exposure. *Nutrients* 12:1795. 10.3390/nu12061795 32560310 PMC7353315

[B35] Pollock D. Alexander L. Munn Z. Peters M. D. J. Khalil H. Godfrey C. M. (2022). Moving from consultation to co-creation with knowledge users in scoping reviews: Guidance from the JBI Scoping Review Methodology Group. *JBI Evid. Synth.* 20 969–979. 10.11124/JBIES-21-00416 35477565

[B36] Portincasa P. Bonfrate L. Vacca M. De Angelis M. Farella I. Lanza E. (2022). Gut Microbiota and Short Chain Fatty Acids: Implications in Glucose Homeostasis. *Int. J. Mol. Sci.* 23:1105. 10.3390/ijms23031105 35163038 PMC8835596

[B37] Sorrenti V. Fortinguerra S. Caudullo G. Buriani A. (2020). Deciphering the Role of Polyphenols in Sports Performance: From Nutritional Genomics to the Gut Microbiota toward Phytonutritional Epigenomics. *Nutrients* 12:1265. 10.3390/nu12051265 32365576 PMC7281972

[B38] Stacy A. Belkaid Y. (2019). Microbial guardians of skin health. *Science* 363 227–228. 10.1126/science.aat4326 30655428

[B39] Thursby E. Juge N. (2017). Introduction to the human gut microbiota. *Biochem. J.* 474 1823–1836. 10.1042/BCJ20160510 28512250 PMC5433529

[B40] Tricco A. C. Lillie E. Zarin W. O’Brien K. K. Colquhoun H. Levac D. (2018). PRISMA Extension for Scoping Reviews (PRISMA-ScR): Checklist and Explanation. *Ann. Intern. Med.* 169 467–473. 10.7326/M18-0850 30178033

[B41] Ursell L. K. Metcalf J. L. Parfrey L. W. Knight R. (2012). Defining the human microbiome. *Nutr. Rev.* 70 S38–S44. 10.1111/j.1753-4887.2012.00493.x 22861806 PMC3426293

[B42] van Veldhoven K. Keski-Rahkonen P. Barupal D. K. Villanueva C. M. Font-Ribera L. Scalbert A. (2018). Effects of exposure to water disinfection by-products in a swimming pool: A metabolome-wide association study. *Environ. Int.* 111 60–70. 10.1016/j.envint.2017.11.017 29179034 PMC5786667

[B43] Wegierska A. E. Charitos I. A. Topi S. Potenza M. A. Montagnani M. Santacroce L. (2022). The Connection Between Physical Exercise and Gut Microbiota: Implications for Competitive Sports Athletes. *Sports Med.* [Epub ahead of print]. 10.1007/s40279-022-01696-x 35596883 PMC9474385

[B44] Xie Y. Wu Z. Zhou L. Sun L. Xiao L. Wang G. (2022). Swimming Exercise Modulates Gut Microbiota in CUMS-Induced Depressed Mice. *Neuropsychiatr. Dis. Treat.* 18 749–760. 10.2147/NDT.S355723 35411144 PMC8994653

[B45] Xu Y. Zhong F. Zheng X. Lai H. Y. Wu C. Huang C. (2022). Disparity of Gut Microbiota Composition Among Elite Athletes and Young Adults With Different Physical Activity Independent of Dietary Status: A Matching Study. *Front. Nutr.* 9:843076. 10.3389/fnut.2022.843076 35369075 PMC8975590

[B46] Zmijewski P. Trybek G. Czarny W. Leońska-Duniec A. (2021). GALNTL6 Rs558129: A Novel Polymorphism for Swimming Performance?. *J. Hum. Kinet.* 80 199–205. 10.2478/hukin-2021-0098 34868429 PMC8607767

